# Recent Progress Towards Quantum Dot Solar Cells with Enhanced Optical Absorption

**DOI:** 10.1186/s11671-016-1457-y

**Published:** 2016-05-23

**Authors:** Zerui Zheng, Haining Ji, Peng Yu, Zhiming Wang

**Affiliations:** Institute of Fundamental and Frontier Sciences, University of Electronic Science and Technology of China, Chengdu, 610054 China; State Key Laboratory of Electronic Thin Film and Integrated Devices, University of Electronic Science and Technology of China, Chengdu, 610054 China

## Abstract

Quantum dot solar cells, as a promising candidate for the next generation solar cell technology, have received tremendous attention in the last 10 years. Some recent developments in epitaxy growth and device structures have opened up new avenues for practical quantum dot solar cells. Unfortunately, the performance of quantum dot solar cells is often plagued by marginal photon absorption. In this review, we focus on the recent progress made in enhancing optical absorption in quantum dot solar cells, including optimization of quantum dot growth, improving the solar cells structure, and engineering light trapping techniques.

## Review

### Introduction

The world energy and environmental crisis urgently calls for development of renewable energies. Among various renewable energy sources, solar energy is abundant and clean. Although solar energy has been an ideal renewable energy, the harvesting of the free and abundant sunshine can be quite costly, which limits the wide deployment of solar power. The next generation of solar cells with high efficiency over 50 % is in urgent need to achieve affordable rates below 0.10 €/kWh (0.14 $/kWh) [1]. In the last 10 years, a lot of efforts have been devoted to low-dimensional structures as building blocks for next generation solar cells [2–7]. Among these nanostructures, the zero-dimensional nature of quantum dots (QDs) with discrete energy levels makes an ideal candidate for intermediate band-based solar cells with a theoretical efficiency of 63 % [8]. Since Luque and Martí proposed the concept of intermediate band solar cell (IBSC), QD solar cells (QDSCs) have attracted great attention and substantial progress has been made in this field [9–14].

Compared with conventional single junction solar cells, an IBSC allows two sub-bandgap photons to create an electron-hole pair via a mid-gap intermediate band. The intermediate energy band introduces additional photon absorption, which in turn contributes to higher photocurrent [8]. The improved utilization of the solar spectrum via intermediate band-assisted transitions to absorb otherwise wasted low-energy photons can largely improve photocurrent and potentially exceed the Shockley–Queisser limit [15–17]. Although the early work has provided solid understanding of the operational principles of IBSCs [18–24], the experimental studies of QD-IBSCs have not achieved any notable improvement in their overall conversion efficiency. QDSCs have often shown improved short-circuit currents compared with the bulk single junction solar cell without QDs, but the overall contribution to efficiency enhancement from the QDs is marginal. Therefore, research efforts in the last 10 years have been mainly focused on improving the photocurrent generation.

In this paper, we review the recent progress made in QDSCs with main focus on the recent effects involving photocurrent enhancement, which has been the major limited to realize high-efficiency QDSCs. A variety of methods used to enhance the optical absorption and photocarrier collection have been reviewed. Finally, this review summarizes the progress of QDSCs with enhanced photocurrent. More comprehensive discussion can also be found in Ref. [14, 25].

### Principles of Quantum Dot Solar Cells

**Fig. 1:**
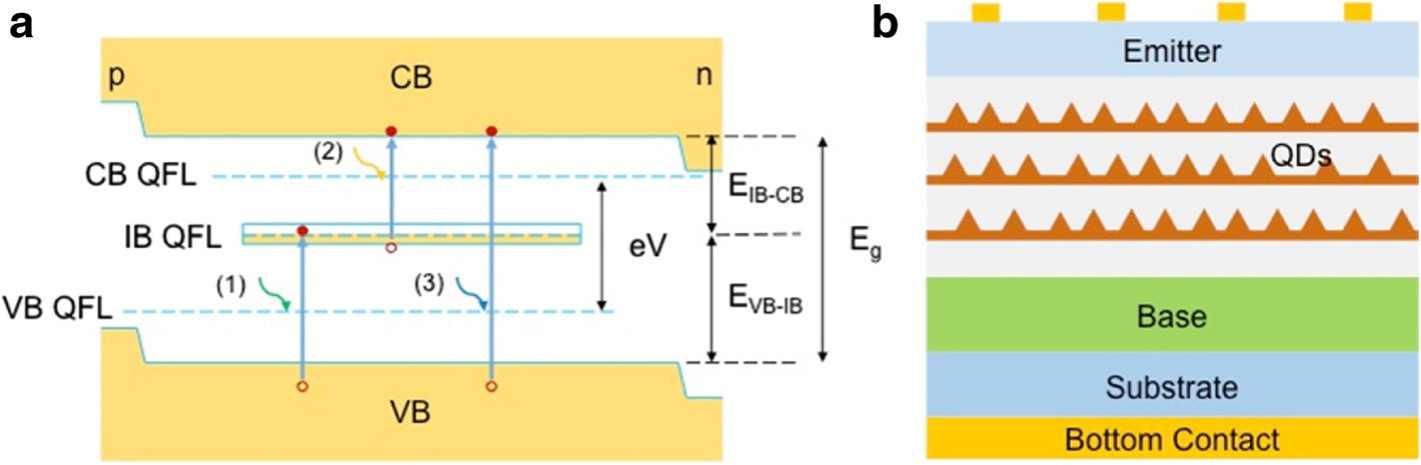
Schematics of (**a**) the band diagram of an IBSC and (**b**) device structure of QD-IBSCs

As schematically shown in Fig. 1a, apart from the conduction band and valence band, the IBSC has an intermediate band in between these two bands for additional absorption of low-energy photons. Electron-hole pairs can be produced by photon absorption via the primary bandgap (VB-CB) as in a conventional single junction solar cell. Additionally, electron-hole pairs can also be generated by optical transitions from valence band to the intermediate band (VB-IB) and then from the intermediate band to the conduction band (IB-CB). The quasi-Fermi level splitting and two-photon absorption preserve the open-circuit voltage as well as generate substantially higher photocurrent. As a result, a very high power conversion efficiency of 63 % is calculated from the ideal IBSC under maximum concentration [8].

QDSCs share same device structures with the quantum well solar cells (QWSCs), which incorporate low-dimensional nanomaterials made from narrow bandgap semiconductors and hence boost the device efficiency by capturing low-energy photons below the primary bandgap. Compared with QWSCs, QDs, instead of QWs, are used at a solar cell junction. The atom-like density of states in QDs not only enables additional photocurrent generation via the discrete energy levels but also preserves the open-circuit voltage [15]. The carrier confinement in all three-dimensions in QDs can enable isolated quasi-Fermi levels which are required to realize of IBSCs [4, 8]. As a result, much higher conversion efficiency is expected from QDSCs compared with QWSCs. Therefore, the unique properties of QDs and the attractive concept of IBSCs have led to intensive research efforts on QD-IBSCs. The research of QD-IBSCs is also largely benefited from the well-established fabrication methods of high-quality QDs in the last couple of decades. Most of the QDSCs adopt a device structure with self-assembled QDs imbedded between the emitter and base of a bulk single junction solar cell, as shown in Fig. 1b. In(Ga)As/GaAs QD system is most used because of its mature fabrication techniques and well-understood optical properties. On the other hand, the transition energies in In(Ga)As/GaAs QDs are quite different from the optimal values for the ideal IBSC, and high-efficiency QDSCs have not been realized yet, although a high theoretical efficiency of 52.8 % is still predicted [26]. Nonetheless, In(Ga)As/GaAs QDSCs have successfully demonstrated the basic operating principles of the IBSCs [25], including splitting of quasi-Fermi levels [2] and QD-mediated two-photon absorption [11, 27]. Therefore, in the last few years, many of the research efforts of QDSCs have been focused on realizing practical QD-IBSCs with high efficiency. In order to achieve this goal, the major challenges associated with QDSCs are yet to be addressed, including recombination in the QDs (radiative and non-radiative), marginal photocurrent collected from the QDs, and degradation of open-circuit voltage [17]. The radiative recombination via the QD intermediate band can be largely suppressed under concentrated light when CB-VB recombination dominates. However, additional non-radiative recombination paths are presented in the QDSCs due to accumulated strain in S-K QDs [28]. To tackle this issue, improvement in QD fabrication and development of new growth techniques have been explored [29–32]. In addition to the strain-induced defects that largely limit the QD absorption volume, the sub-bandgap absorption in QDSCs is rather low and only contributes to ~1 % of the overall efficiency [17]. Moreover, the slightly improved photocurrent has been largely undermined by the voltage loss as a result of thermal coupling of the QD states and the continuum states [10, 30, 33]. Therefore, the major research activities have been focused on addressing these challenges facing QD-IBSCs. The following sections will review the recent efforts to achieve practical high-efficiency QDSCs through improving photocurrent.

### Recent Efforts to Improve Photocurrent of QDSCs

Although the addition of QDs in a single junction solar cell normally shows additional photocurrent, improvement in short-circuit current is well below the expectation for high-efficiency solar cells. The marginal improvement in the device efficiency with QDs is largely attributed to the non-radiative recombination, low QD absorption volume, and low optical transition rate [34]. In order to obtain high photocurrent, both the QD material quality and device structure have to be optimized. Moreover, photonic structures can also be used to boost the light absorption in the QDSCs. Here, these efforts are summarized.

#### Optimization of QDs

**Fig. 2:**
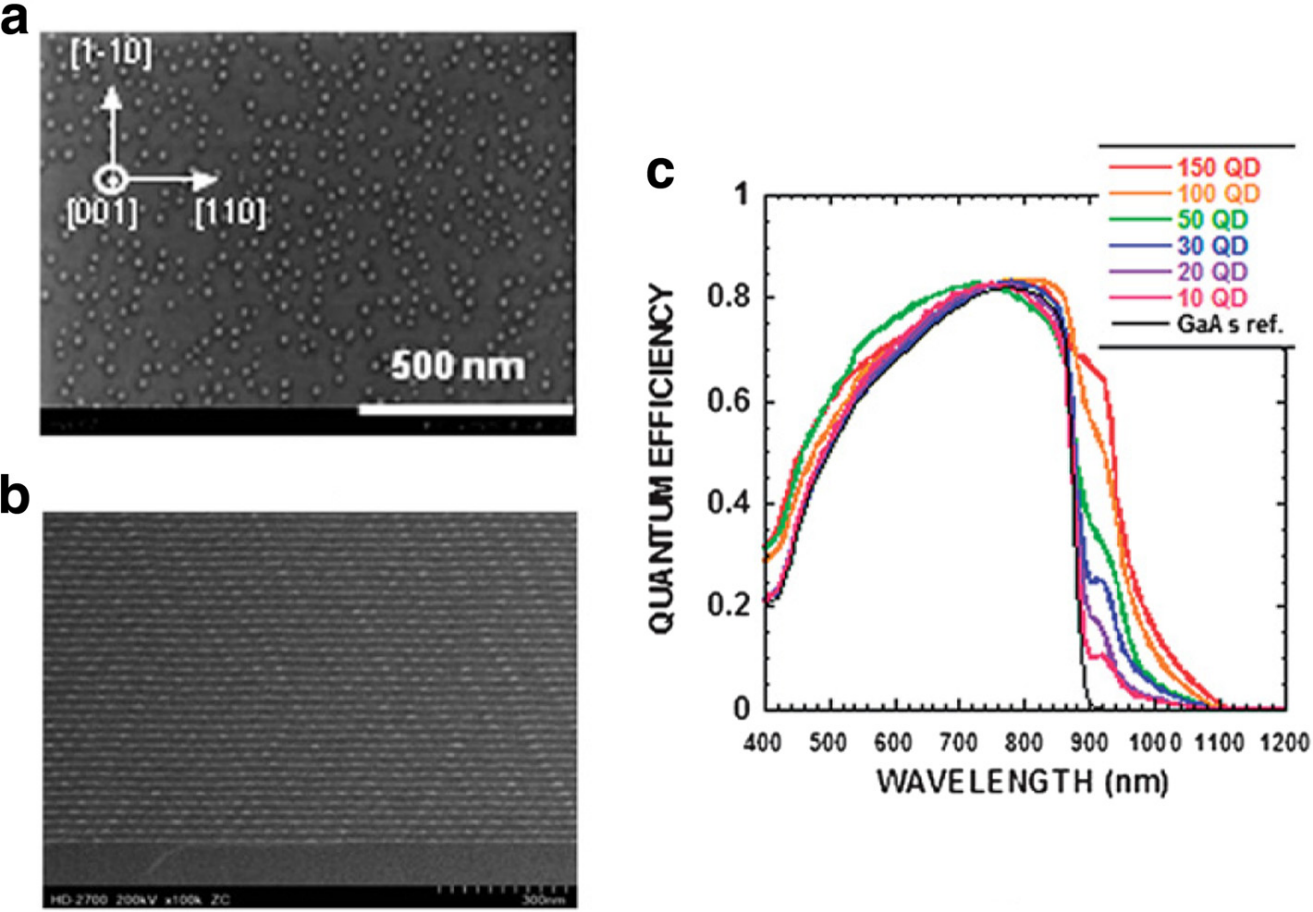
**a** SEM micrographs of the surface plane on top of 400-stack In_0.4_Ga_0.6_As QD structures. The ultra-high stacked structures have good surface morphologies even after the stacking of 300 or 400 QD layers. **b** Enlarged cross-sectional STEM images of bottom portions of 300-stack In_0.4_Ga_0.6_As QD layers. No dislocations were generated after the stacking of 300 layers, even though no strain balancing was employed during the growth. **c** EQE spectra of multi-stacked In_0.4_Ga_0.6_As QD solar cells and a GaAs reference cell. The EQEs of the 10-, 20-, 30-, 50-, 100- and 150-stack In_0.4_Ga_0.6_As QD solar cells are indicated. Reproduced from Ref. [12] with permission from The Royal Society of Chemistry

A straightforward way to improve short-circuit current is to increase the absorption volume of QDs. Multiple stacked In_0.4_Ga_0.6_As/In_0.2_Ga_0.8_As (In_0.4_Ga_0.6_As) QDSCs with 50 (30) layers of QDs have shown distinct improvement in short-circuit current density [35, 36]. Using similar method, highly stacked In_0.4_Ga_0.6_As QDs up to 400 layers were also reported. Although improvement in short-circuit current has also been observed from QDSCs with up to 150 layers of QDs, significant degradation in open-circuit voltage results in degradation of the overall device efficiency [12, 35], as shown in Fig. 2.

The difficulty to increase the absorption volume QDs, e.g., the number of QD layers, is that the accumulated strain generates various types of defects and largely undermines the improvement of photon absorption [22, 37]. To minimize the number of strain-induced defects that are deleterious to both optical and electronic properties, strain-compensation layers are deposited for multiple stacked QDSCs [38]. By using GaP strain compensation layers, InAs QDs with good structural and optical properties up to 50 layers have been reported [39]. The improved material quality has also led to increase in short-circuit current and reduced dark current [40]. Additionally, the reduced strain-induced defects also decrease non-radiative recombination, and then, high open-circuit voltage can be obtained [10]. Bailey et al. reported 0.5 % enhancement in absolute efficiency from a 40-layer QDSC with reduced InAs coverage and GaP strain compensation layers compared with the GaAs reference cell [30].

A number of different materials have also been explored to improve QD quality. Highly stacked QDs up to 100 layers are also achieved by using dilute nitride GaAsN strain compensation layers [41, 42]. The effectively compensated strain results in a distinct improvement in short-circuit current as high as 2.47 mA/cm^2^ [41]. Strain-balanced In_0.47_Ga_0.53_As/GaAs_1 − x_P_x_ QDs have also been reported with improved quality as well as uniformity on GaAs (311) substrates [29]. Furthermore, strain-compensated InAs/GaNAs QDs with additional strain-mediating GaInNAs layers can not only shift the absorption to long wavelength but also increase the surface density of QDs [43]. Strain reducing layers is also beneficial for realizing high-performance QDSCs. It has also been reported that an addition of Ga_0.90_In_0.10_As strain-reducing layers in an InAs/GaAs QDSC results in a 1.19 % improvement of the conversion efficiency of a GaInP/Ga(In)As/Ge triple junction solar cell due to reduced Shockley–Read–Hall recombination centers [44].

**Fig. 3:**
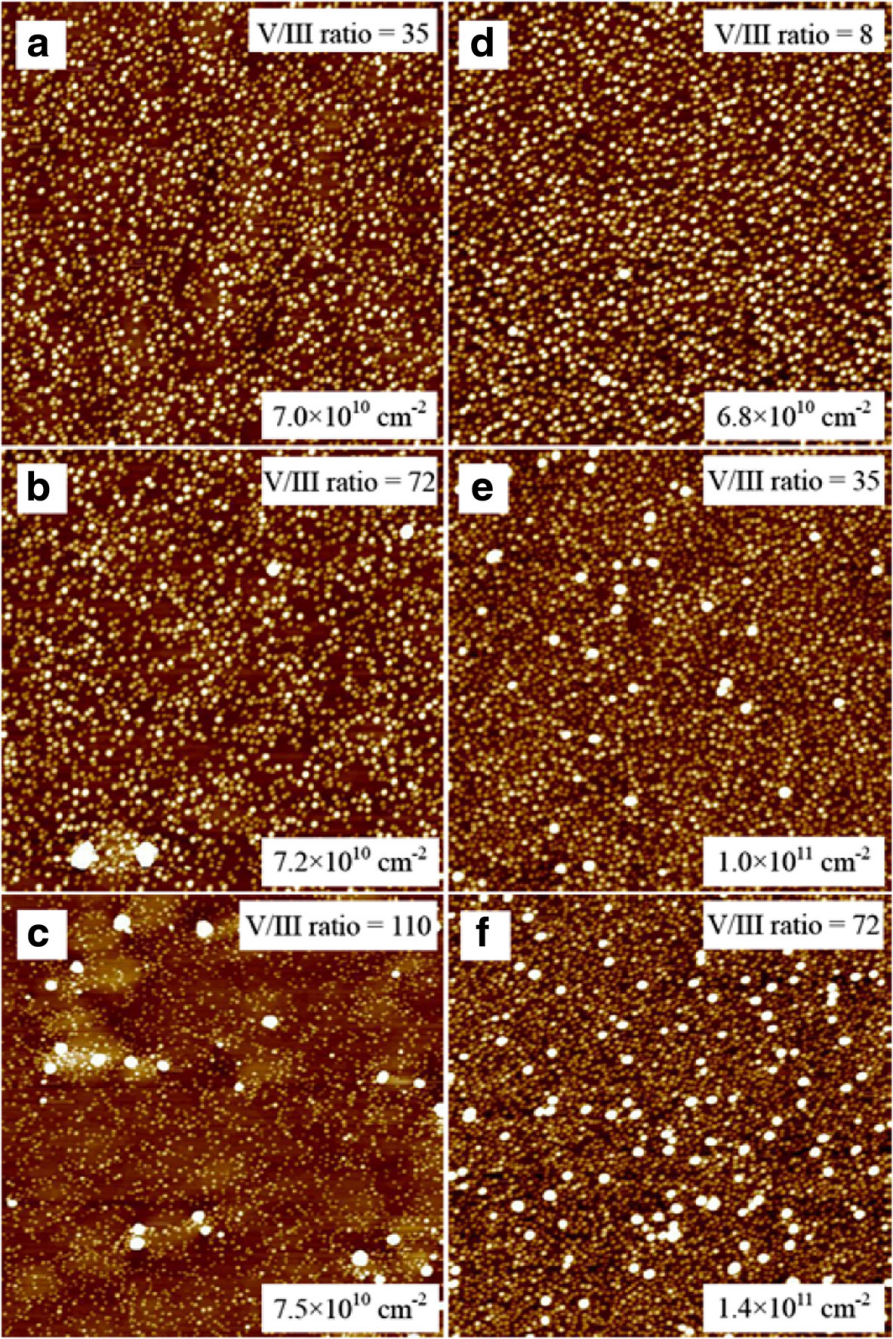
2 × 2 μm^2^ top view AFM images of 2.0 ML InAs QDs with V/III ratios of (**a**) 35, (**b**) 72, and (**c**) 110; 2.8 ML InAs QDs with V/III ratios of (**d**) 8, (**e**) 35, and (**f**) 72. Reprinted from Ref. [45] with the permission of AIP Publishing

Another effective way to increase the absorption volume is to increase the surface density of QDs. In Fig. 3, a QDSC with a high sheet density of 7.0 × 10^10^ cm^−2^ was obtained via optimization of growth temperature and V/III flux [45]. Despite the high QD density, the formation of defective QDs, e.g., In segregation, resulted in poor short-circuit current [45, 46]. Sb-mediated growth was capable of achieving high QD density over 1 × 10^11^ cm^−2^ with a low density of defective QDs, which thus led to a distinct enhancement in short-circuit current [47]. Apart from the Stranski–Krastanov (S-K) QDs, high-density QDs can also be obtained by using another growth mode. Submonolayer (SML) QDs have been reported to have high areal density (~10^11^ cm^−2^), adjustable aspect ratio, uniform size distribution of QDs, and absence of wetting layer [48, 49]. By using InGaAs/GaAs SML QDs, the solar cell has shown improved performance compared with an InGaAs/GaAs quantum well solar cell of the same structure [48]. Similar to S-K QDs, SML QDs can also significantly contribute to photocurrent enhancement. Kim et al. recently demonstrated an improved short-circuit current of the InAs/GaAsSb SML QDSC compared with the reference GaAs solar cell [50]. Also, an InGaAs/GaAs SML QDSC is also demonstrated with better short-circuit current than the reference S-K QDSC [49]. It should be noted that SML QDs show a higher compressive strain and thus more non-radiative recombination centers than S-K QDs [49]. Nonetheless, the high areal density of QDs can compensate the non-radiative recombination centers generated. In combination with strain compensation technique, further improvement in short-circuit current can be expected.

Apart from the strained S-K QDs and SML QDs, quantum structures grown by different modes can be used as promising alternatives for improving photocurrent. Quantum well dots (QWD), two-dimensional layers with lateral modulation of thickness and composition, have unity surface coverage, which facilitates higher absorption as compared with S-K QDs and demonstrates significantly improve sub-bandgap photocurrent [51]. Strain-free quantum structures fabricated by droplet epitaxy have also show promise in boosting photon absorption [52–55]. Based on these strain-free nanostructures grown by droplet epitaxy, additional photocurrent was clearly demonstrated [56–59]. Although further efforts to improve material quality are needed, the two-photon absorption observed in strain-free QDSCs opens new opportunities for QD-based high-efficiency intermediate band solar cells [59, 60].

#### Optimization of Device Structures

**Fig. 4:**
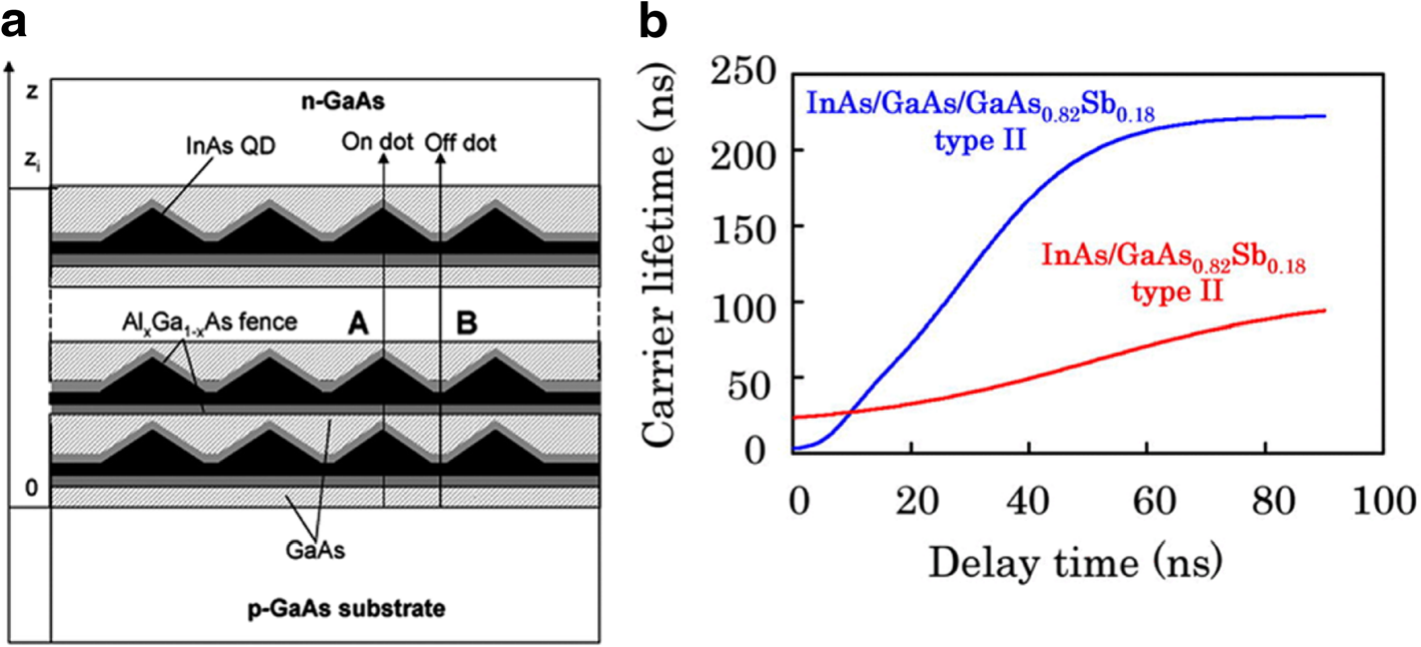
**a** Structure of the quantum dots in a fence barrier (DEFENCE) solar cell. Reprinted with permission from Ref. [62] Copyright (2007) American Chemical Society. **b** Time evolution of carrier lifetime for the two samples with type II band structures. Excitation intensity is 38 mW/cm^2^. Reprinted from Ref. [77] with the permission of AIP Publishing

In addition to increase absorption with more QDs, engineering the QD structures also plays a critical role in boosting the photocurrent. For example, through simple truncation of the dot height, an increase in both short-circuit current density and open-circuit voltage has been observed as a result of improved photocarrier extraction and reduced carrier recapture probability by the QDs [61]. To boost photon absorption, Wei et al. proposed a quantum-dot-in-a-fence (DFENCE) structure which consists of InAs QDs enclosed by thin Al_x_Ga_1 − x_As “fence” layers of larger energy bandgap [62], as shown in Fig. 4a. The fences facilitate sub-bandgap photocarrier generation rather than recombination in the QDs, and hence, a very high solar power conversion efficiency of 45 % can be expected for InAs QDSCs with Al_x_Ga_1 − x_As “fence” layers under AM1.5 conditions. Experimentally, such structures have not shown any clear improvement in device performance yet, but the thermal extraction of carriers was suppressed due to improved quantum confinement [63].

Engineering the QDs locally to change the carrier dynamics can also lead to a higher short-circuit current. A simple but effective way to achieve this goal is doping in the QD region, which has been reported to reduce non-radiative recombination via defect passivation [64] and to improve the photocarrier collection by build-in field [65]. The doping in the QD region forms charged QDs that also reduce the probability of electron capture. Although state filling can also decrease interband quantum dot absorption [66], the charged QDs enhance the collection of photocarriers generated above bandgap and lead to overall improvement in photocurrent [67, 68]. It has also been shown that the positioning of the QD layers can also largely affect the performance of QDSCs [69], which also reflects the effects of doping [70].

Substantial efforts have also been made to type II QDs to improve short-circuit current [20, 71–75]. QDSCs can benefit from largely enhanced absorption coefficient, particularly for transitions from extended states to bound states, by using type II QDs rather than type I QDs [76], as depicted in Fig. 4b. Yet, it is still needed to find new material system with even high absorption coefficient to compete with the higher bound-to-bound state absorption coefficient in type I QDs. Another attractive feature of the type II QDSCs is the extremely long radiative lifetime over 200 ns [77]. Such a long carrier radiative lifetime facilitates the photocarrier collection as long as non-radiative recombination centers are suppressed with the presence of additional strain [78]. Moreover, the reduced Auger recombination rate in type II structure can also benefit the QDSC performance [72].

#### Light Trapping

A very interesting and promising method to improve photocurrent of QDSCs is light trapping. To full fill the promise of QDSCs, the QD density needs to be significantly improved (>1000). Such a requirement poses a significant challenge for material growth. If the optical path can be improved, high density of QDs is not necessarily required [24]. For example, given a QD density achievable by existing growth techniques, an optical absorption enhancement over 50 can potentially realize high-efficiency QDSCs beyond the Shockley–Queisser limit [24].

**Fig. 5:**
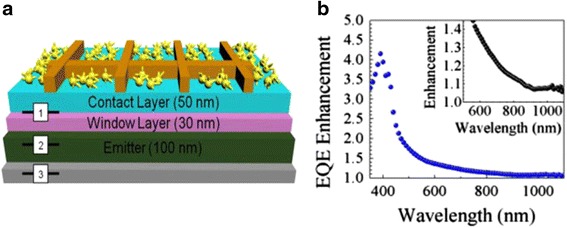
**a** Schematic illustration shows the depth profile positions that were used for the FDTD simulation of enhanced optical absorption in the QD solar cell with deposition of gold nanostars. **b** The EQE enhancement ratio after depositing gold nanostars. The *inset* shows the EQE enhancement ratio in the long-wavelength range. Reprinted from Ref. [82], Copyright 2015, with permission from the Elsevier

Plasmonic structures can be an effective way to enhance the optical absorption in QDSCs. It has been shown that it is possible to obtain an absorption enhancement factor up to ~300 by using the strong scattered near-field potential from metal nanoparticles [79]. Although metallic nanoparticles cannot be placed in close proximity to QDs without undermining the material quality, surface nanoparticles can be used as good light scatter to improve optical path in QDSCs [80]. The effective forward scattering of metal nanoparticles deposited on QDSC surface has shown distinct improvement in short-circuit current [81]. Using similar technique but with novel metal nanoparticles, e.g., nanostars, a broadband enhancement in photon absorption has been observed in QDSCs [82], as illustrated in Fig. 5. Especially, external quantum efficiency in short-wavelength region has been improved by fourfold. The enhancement is originated from both the near-field enhancement and effective light scattering. It also demonstrates that appropriate control of shape, size, and density of the metallic nanoparticles plays a critical role in achieving panchromatic photon absorption. However, the surface plasmonic structures do not show clear improvement in absorption in the QD region. By inserting a TiO_2_ between the QDSC and metal nanoparticles, the plasmon resonance wavelength was red-shifted to the QD wavelength region [83]. As a result, a pronounced improvement in long-wavelength photon absorption has been achieved in the QDSCs with TiO_2_/Ag back reflector and leaded to 5.3 % enhancement in short-circuit current. Back reflector has also been developed by growing a brag reflector beneath the QDSC. A brag reflector centered at 920 nm leads to about ~2 % increase in short-circuit current due to enhanced absorption in the long-wavelength region [84]. As a result, a maximum efficiency of 24.93 % (AM 1.5D, 30 suns) has been obtained from the QDSCs with brag reflector, which is nearly as high as the efficient GaAs reference cell (25.75 % at AM 1.5D, 10 suns). Interestingly, an epitaxial lift-off QDSC thin film can act as a resonance cavity by itself [85]. In addition to the enhancement of photon absorption in the QDSC film, there is no need for additional processing steps to create photonic structures, which is desired in terms of reducing cost. Further development and optimization of photonic structures will enable substantial improvement of solar energy harvesting by using QDs.

## Conclusions

In the present paper, we have briefly reviewed the efforts to improve the photocurrent in QDSCs. A number of different methods have so far been examined to improve the optical absorption as well as photocarrier collection in QDSCs. Although each of these methods shows promise in boosting the cell performance in terms of photocurrent, there is still a lot of room to improve. Till now, the absorption from the QDs is still much inferior to the bulk absorption. Undoubtedly, novel designs and further improved growth of QDSCs need to be in place to achieve efficiency exceeding that of single junction solar cells. Nonetheless, the progress made so far discussed here, including growth of high-density QDSCs, modification of carrier dynamics, and light trapping, provides helpful guidelines for further development of high-efficiency QDSCs.
